# Physiological Action of Obtundents

**Published:** 1891-09

**Authors:** L. E. Custer

**Affiliations:** Dayton, Ohio.


ARTICLE II.

### PHYSIOLOGICAL ACTION OF OBTUNDENTS.

BY L. E. CUSTER, D. D. S., DAYTON, OHIO.

Read before the American Dental Association, Saratoga Springs, N. Y., August 4, 1891.

This paper has been written with a view to making clear and harmonizing a few conflicting ideas regarding the action of obtundents of the sensitiveness of the dentinal fibril.

Owing to conflicting theories, it will be fair to assume that this tissue whose sensibility we desire to obtund is of the nature of simple protoplasm, with, in this instance, an exalted sensitive function. It contains albumen, and this is therefore coagulable; it is made up largely of water, and may be desiccated; it is incased in a tube, and when it does not entirely fill this tube a fluid fills the remaining space. This fluid may be removed, and the temperature of the fibril may be reduced. These are the conditions upon which we are to work.

The dentinal fibril does not possess a blood circulation, and on that account the nutritive movements are slow; so slow that any agent which produces local anæsthesia in other parts in a few minutes will be very slow in acting to any depth in the dentinal fibril; so slow, indeed, that we need hardly look in this direction for a practical agent. Cocaine as a typical representative of this class of agents has not been effective for this reason. Any agent which is to act upon the sensitive function of the dentinal fibril must be more powerful than cocaine; it must not coagulate albumen; it must have an affinity for water; it must possess a penetrating property, and insinuate itself into the tubule, for it will not be carried in by circulation and but very slowly by any nutritive movement. Until we find an agent possessing these qualities, we need not expect to obtund sensitive dentine by suspending the fibril's irritability.

The most satisfactory results have been obtained in other directions. The dental fibril has a definite composition. The elements of all the proteids are in a definite proportion, and we have reason to believe that the proportion is very delicately balanced. If the structure of the fibril be changed, its irritability ceases and it can no longer communicate sensation.

The change of structure most easily accomplished is the coagulation of its albumen and the withdrawal of its water. If the albumen is coagulated, it is as effectual in checking neural movement as though the albuminous ingredient had been withdrawn; indeed, the coagulation of albumen itself renders it harder, and its presence would prevent any exhibition of life more readily than if it were removed.

The coagulation of albumen, unless produced by heat, is somewhat self-limiting, so that since heat of 160° F. is not allowable for sensitive dentine, the action of present known coagulants is not entirely sati factory. Before coagulation can be a practical success, we must have an agent which penetrates to some depth.

Penetrating escharotics, such as arsenic and the like, since they burn the tissue beyond recovery and endanger the pulp, should not be considered.

The other method of changing the structure of the dentinal fibril is by the removal of the water. The water holds a twofold relation with the fibril,—that which is a constituent of the fibril itself, and that which fills any inequalities between the fibril and Neumann's sheath. One is a constituent, and the other a condition. The water which is a constituent is always present; it is always found where life and motion occur; it is always a necessary condition for neural activity; it makes up three-fourths of the entire body and about ninety per cent. of the dentinal fibril. Constituting such a large proportion of the fibril, it is evident that there would be a proportionate change in the structure and physical character if the water were removed. The size would be decreased, the albumen if coagulated would become harder, and in this desiccated condition it would be practically dead, incapable of performing nutrition and function.

The water which fills any inequalities between the fibril and its investments may be accessory in the transmission of sensation. In this relation it may transmit vibration to the fibril or even to the fibriloblast. One of the most delicate nerves of special sense, the auditory, receives its impressions through the endolymph in which its terminals float. So if the water surrounding the fibril should be removed, the fibril's transmitting power may be affected.

The watery contents of the tubule are withdrawn by evaporation and by bringing an agent in contact with it for which it has an affinity. Evaporation is produced by raising the temperature and subjecting to a current of air. This is practically accomplished by the repeated blasts from a hot-air syringe. The water surrounding the fibril is comparatively easily absorbed, but to loosen the molecular grip in the fibril itself requires more force. There are many agents which have an affinity for water, but not all are suitable for use in the cavity of a tooth. The agents most desirable for this purpose would be alcohol or glycerin. Alcohol possesses a property different from glycerin which makes it valuable: it is volatile. Water is taken up by the alcohol and evaporates with it. Of course the affinity is increased the nearer the alcohol is absolute.

The water is more thoroughly withdrawn when the two principles, affinity and evaporation, are employed together.

As compared with coagulation as a means of changing the structure, dehydration is more thoroughly accomplished with the present known methods, and hence the results are more satisfactory; so much so that I suppose the majority of operators use dehydration almost entirely as a means of obtunding sensitive dentine.

If we use a combination of the two methods of change of structure,—desiccation followed by coagulation,—we will secure more profound results. As a proof of the truth of this statement, we have an agent which when used on sensitive dentine both withdraws the water and coagulates the albumen, and a more effective single medicament for sensitive dentine we have not. This stands alone and at the head of the list as a practical, efficient, and I may say safe agent as an obtundent. I refer to chloride of zinc,—not a fluid when its affinity for water has been satisfied, but a crystal which will deliquesce when placed in the cavity.

The last method by which the fibril's transmitting power may be lessened is by changing the temperature. This is of value as an obtundent of sensitive dentine on the same principle that it acts upon the soft tissues. As a condition for the perfect performance of nerve-function, there must be maintained a certain temperature which is normal to the organism. If that temperature is lowered, neural activity is lessened somewhat proportionate to the departure from normality, so that there may be a point reached where complete anæsthesia is produced. Of all the obtundents of sensitive dentine, extreme cold is the most effective; not that it is any better than perfect coagulation or dehydration, but because the temperature of dentine may be reduced more thoroughly than the albumen may be coagulated or the water withdrawn in ordinary practice. This is because of the distance of the bulbous portion of the pulp from collateral circulation, there being no blood circulation in the dentine, the ease with which the crown of the tooth may be isolated from surrounding sources of warmth, and also because we have refrigerating agents so simple and effective in their action that they may be made to act even beyond the necessities of the case.

The reduction of temperature is best accomplished by the use of volatile agents, such as sulphuric ether, chloride of methyl, rhigolene, or nitrous oxide.

It has been claimed for ether and nitrous oxide when used upon dentine that they act by abstracting the moisture. If we substitute the word *heat* for *moisture*, their action will be correctly stated. It is a mistake to attribute the action of the above agents to any water which they may withdraw, for these are cold-producing agents, and it is contrary to the laws of the physics to produce evaporation by lowering the temperature; so that water cannot be withdrawn by these agents unless there is an affinity, which with ether there is none, and with nitrous oxide the temperature is lowered so rapidly that the water is frozen before any quantity could be removed by affinity. Any agent which reduces the temperature opposes evaporation or any chemical action upon the tubular contents. All such agents obtund sensitiveness in but one way, and that is by reducing the temperature.

It will be observed from the foregoing that the function of the fibril may be completely suspended by coagulating all its albumen, by withdrawing all its water, or by lowering its temperature to a certain point. But the entire coagulation of its albumen is practically impossible at present, the entire removal of its moisture almost impossible, and the reduction of temperature is the only one which acts to any desired depth. So theoretically, and it has been found practically, that, except arsenical preparations, refrigerating agents are the most effective obtundents of sensitive dentine allowable.

Recently there have been introduced three new methods having the same principle of action which are misleading. The thermal obtunder of Small, the Milton obtunder, and the Richmond obtunder are instruments and methods which use an elevation of temperature and the vapor of an alcohol or an essential oil, or a combination of these, upon the dentine. There is a silent claim in all these that there is a specific action of the vaporized agent upon the conductivity of the fibril, otherwise why use anything but the jet of hot air itself?

The effect of throwing a warm or hot blast upon a tooth is to heat it. By heating the dentine evaporation of its moisture takes place. The fibril dries up, and there is an outward movement as the water vaporizes and escapes from the tubule. No medicine can possibly enter the tubule during this time unless it has an affinity for water. When an oil or any agent which has no affinity for water is used in these methods, it will be apparent how utterly useless it is. Then the oil is not applied in its liquid state, but in a vapor. Is the vapor of the oil of cassia more powerful than the liquid form in preventing the development of micro-organisms? What virtue can possibly be attached to attenuation, that the liquid must be vaporized? Power lies in concentration. What effect can a vapor have upon the dentinal fibril if it simply fills the cavity of the tooth? In order to be effective it must condense and enter the tubule before it can come in contact with the fibril, and this cannot take place when it is applied with heat.

The application of a vaporized agent in a warm or hot blast is essentially one of *desiccation*. The heat is the only virtue in this unless the agent has an affinity for water, when it may aid in carrying off the water vapor. The vapor of alcohol is effective on this account, and I think those who have recommended the use of oils or any agent which has no affinity for water are laboring under a delusion.

Unfortunately, the agents which are most effective are most dangerous to the pulp, and in our selection of an agent for the case at hand this is to be borne in mind.—*Dental Cosmos.*

---

ARTICLE III.

### STERILIZATION OF DENTAL INSTRUMENTS.

